# Association Between Breastfeeding and Neurodevelopment at 6 Years of Age in the French PELAGIE Birth Cohort

**DOI:** 10.1111/jmwh.13766

**Published:** 2025-06-03

**Authors:** Marion Monperrus, Cécile Chevrier, Nathalie Costet, Maela Le Lous, Jonathan Y. Bernard, Gaïd Le Maner‐Idrissi, Agnès Lacroix, Pauline Blanc‐Petitjean, Sylvaine Cordier, Florence Rouget, Christine Monfort, Ronan Garlantézec, Rémi Béranger

**Affiliations:** ^1^ Department of Obstetrics and Gynecology University Hospital Rennes Rennes France; ^2^ Centre d'investigation clinique de Rennes (CIC 1414), INSERM University Hospital Rennes Rennes France; ^3^ Univ Rennes, CHU, INSERM, EHESP IRSET (Institut de recherche en santé, environnement et travail) Rennes France; ^4^ Department LTSI ‐ INSERM UMR 1099 University of Rennes Rennes France; ^5^ Université Paris Cité and Université Sorbonne Paris Nord INSERM, INRAE, Centre for Research in Epidemiology and StatisticS (CRESS) Paris France; ^6^ Université Rennes 2 Laboratoire de psychologie: comportement, cognition et communication (LP3C) Rennes France

**Keywords:** breastfeeding, cohort, cognition, child development, neuropsychology

## Abstract

**Introduction:**

Breastfeeding has been shown to be associated with improved child cognitive performance, but the causality of this association is still debated because it tends to disappear when accounting for maternal cognitive performance and socioeconomic status. We aimed to explore the relationship between breastfeeding and the cognitive performance of children in the French general population.

**Methods:**

From the PELAGIE woman‐child cohort, which included pregnant women between 2002 and 2006 in Brittany (France), 286 children were evaluated using the Wechsler Intelligence Scale for Children (WISC‐IV) and the Developmental Neuropsychological Assessment (NEPSY) scales at age 6. Associations between breastfeeding and cognitive performance were assessed using multivariable linear regression models adjusted for maternal verbal cognitive performance and education level, familial stimulation and environment (Home Observation for Measurement of the Environment scale), and Rey's Social Deprivation Index (contextual indicator). In addition, we performed structural equation modeling (SEM) to investigate the complex interrelation between these variables.

**Results:**

Children who were breastfed for at least 4 months had significantly higher scores on the WISC Verbal Comprehension Index (WISC‐VCI) than those who were never breastfed or who were breastfed less than 15 days (β_adjusted_, 4.95 points; 95% CI, 0.54‐9.37). Among the 193 children who were breastfed, the duration of breastfeeding, in particular during the first 4 months, was increasingly associated with the WISC‐VCI score. We also observed statistically significant associations between breastfeeding itself or the duration of breastfeeding and better performance on several NEPSY subtests, including visual attention, design copying, arrows, and narrative memory. SEM analysis confirmed these associations. No statistical association was observed between breastfeeding and the WISC Working Memory Index or other NEPSY subtests.

**Discussion:**

These data support current national and World Health Organization global 2025 targets of promoting breastfeeding for at least 4 to 6 months.

## INTRODUCTION

The World Health Organization (WHO) recommends exclusive breastfeeding up to 6 months old for infants to reduce child mortality worldwide by limiting the risk and impact of bacterial and viral infections. In high‐income countries, where health conditions and access to health care are of higher quality, the goal of breastfeeding focuses principally on better child development, immune transmission, prevention of childhood diseases, and a reduced risk of metabolic diseases (eg, obesity and diabetes) in later life.^1^, ^2^, ^3^, ^4^ The WHO guidelines for Europe, therefore, recommend the exclusive breastfeeding of all infants from birth to the approximate age of 6 months, and at least for the first 4 months of life.^4^ In the United States, the American Academy of Pediatrics recommends exclusive breastfeeding for approximately 6 months after birth, and continued breastfeeding for up to 2 years, depending on the woman's choice.^5^

**QUICK POINTS**

✦ A longer duration of breastfeeding is linked to better performance in various neurocognitive domains in 6‐year‐old children.
✦ No threshold effect was found, but cognitive gains were greatest in the first 2 to 6 months of breastfeeding.
✦ The association between the duration of breastfeeding and cognitive performance scores was stronger in economically‐deprived areas, suggesting breastfeeding may help counteract adverse environmental factors.
✦ Robust analyses, such as structural equation modeling, strengthen confidence in the link between breastfeeding and child neurodevelopment.
✦ Implementing breastfeeding support programs for families in precarious situations is crucial because the benefits of breastfeeding, such as improved neurodevelopment, may help break the cycle of poverty.

The relationship between breastfeeding and cognitive outcomes in children has been extensively explored since the first known report in 1929.^6^ A randomized trial conducted in Belarus in 1997 by Kramer et al reported that prolonged and exclusive breastfeeding improved cognitive development at 6.5 years, with a higher intelligence quotient (IQ) when perinatal patients were encouraged to breastfeed (modeled on the Baby‐Friendly Hospital Initiative of the WHO and UNICEF).^7^ Several observational studies have reported a higher IQ among breastfed children, with quantifiable effects persisting until and throughout adulthood.^8^, ^9^, ^10^, ^11^, ^12^, ^13^, ^14^, ^15^ However, others did not show any difference.^16^, ^17^, ^18^ The studies that showed no difference tended to adjust their analyses for socioeconomic status and maternal cognitive performance, suggesting that the positive effect of breastfeeding on neurodevelopment may have been because of residual confounding. Moreover, no study has simultaneously explored the relationship between breastfeeding and scores on various neurodevelopmental scales, considering the interrelation between different scales that might be correlated.

We aimed to assess the association between breastfeeding and cognitive performance among six‐year‐old children from the woman‐child cohort PELAGIE (Perturbateurs endocriniens, Étude Longitudinale sur les Anomalies de la Grossesse, l'Infertilité et l'Enfance; Endocrine Disruptors, Longitudinal Study on Pregnancy Disorders, Infertility, and Childhood Development), taking into account known confounding factors such as individual and contextual socioeconomic levels, maternal IQ, and child stimulation within the family environment (ie, the level of parental support in the early cognitive and socioemotional development of the child). In addition, for the first time, we aimed to stratify the scores obtained with the different scales by the social deprivation index and simultaneously analyze them by structural equation modeling (SEM) to investigate the complex interrelations among these variables.

## METHODS

### Study Population

The PELAGIE woman‐child cohort has been described elsewhere.^19^ Briefly, the cohort continuously enrolled 3421 pregnant women from Brittany, France, between 2002 and 2006. Pregnant women were recruited before 19 weeks gestational age by their gynecologist, obstetrician, or sonographer at the first pregnancy visit. When the children were 6 years old, 591 woman‐child pairs were randomly selected from the live‐born singleton children for neurodevelopmental assessment. Twenty were excluded because of preterm birth (<35 weeks’ gestation), neonatal respiratory distress, hospitalization, or Down syndrome. A further 125 could not be reached by telephone, and 18 were excluded because the child had previously undergone neuropsychological or behavioral tests. Among the remaining woman‐child pairs, 287 (67%) women agreed to participate with their child in the neuropsychological follow‐up. One woman‐child pair was further excluded for missing values on breastfeeding variables. Women were asked to complete a self‐administered questionnaire at inclusion and the second and sixth birthdays of the child. The questionnaires included information about sociodemographic characteristics and maternal lifestyle factors, as well as the behavior and activities of the child. Breastfeeding status (yes or no) and duration were primarily determined using data collected when the child was 2 years old (198 women, 69%). When missing, data were imputed based on data provided when the child was 6 years old. All women provided written informed consent. Children also provided verbal assent at 6 years of age. This study was approved by the French Consulting Committee for the Treatment of Information in Medical Research (number 09.485) and by the French National Commission on Information Technology and Liberties (number 909347). This report followed the Strengthening the Reporting of Observational Studies in Epidemiology (STROBE) guidelines (Supporting Information: Appendix S1).

### Neurodevelopment Assessment

When children turned 6 years old, 2 trained psychologists conducted neurodevelopmental assessments of families at home: one performed the child's tests, and the other assessed the IQ and family environment of the women. Two domains of the Wechsler Intelligence Scale for Children, fourth edition (WISC‐IV) were used to assess the children's cognitive performance: 3 subtests of the Verbal Comprehension Index (VCI; similarities, vocabulary, and comprehension) and 2 subtests of the Working Memory Index (WMI; digit span and letter‐number sequencing).^20^ Scores were normalized on a scale with a mean of 100 and a SD of 15 (range, 40‐160). Higher scores indicate better cognitive performance. The neurodevelopmental assessment was performed using 8 subtests in 3 domains of the Developmental Neuropsychological Assessment, first and second editions (NEPSY) battery: attention and executive functioning (3 subtests: towers, auditory attention and response set, and visual attention), visuospatial processing (2 subtests: design copying and arrows), and memory and learning (3 subtests: memory for faces, memory for names, and narrative memory).^21^ Overall, the NEPSY provides information about possible childhood neurological, developmental, and learning disabilities. For each subtest, the mean scaled score was 10, and the SD was 3 (range, 1‐19). Higher scores indicate better cognitive performance. The Wechsler Adult Intelligence Scale, third revision (WAIS‐III) was used to document the women's verbal IQ.^22^ Higher scores indicate better verbal cognitive performance. The Home Observation for Measurement of the Environment (HOME) inventory allowed evaluation of the quality and extent of stimulation available to the child in the familial environment.^23^ A higher HOME inventory score indicates a more supportive and stimulating home environment.

### Potential Confounding Variables

We identified potential confounders using a directed acyclic graph (DAG) based on the literature and by consensus among the authors (Supporting Information: Appendix S2).^24^ We used the same set of covariates in all statistical models: the woman's verbal IQ (WAIS), education level, familial stimulation, and environment scale (HOME score), Rey's Socioeconomic Deprivation Index (neighboring socioeconomic deprivation score, tertiles based on the Brittany population),^25^ infant birth weight, parity, mode of birth, and occupational activity during pregnancy.

### Statistical Analyses

We first categorized the duration of breastfeeding into 3 classes: no breastfeeding or early weaning of the newborn (≤15 days), duration of breastfeeding from 16 days to 4 months (median value for breastfed children in the study population), and duration of breastfeeding greater than 4 months. We grouped children who were weaned early and who were never breastfed according to the evolution of human milk composition, from colostrum to transition milk after 5 days, and from transition milk to mature milk after 15 days.^26^ Then, among breastfed children only, the duration was considered continuously. These data were log‐transformed (log_10_) to improve the model fit statistics, such as the Akaike information criterion, used in our statistical analysis. For participants for whom the scoring was incomplete for the WISC‐VCI (n = 4), WISC‐WMI (n = 12), or WAIS (n = 2), we predicted the missing scores from their available subtests using a linear regression model (except for 4 children with missing data for both WISC‐WMI subtests). One to 3 children were excluded from the analyses for the 3 NEPSY subtests due to missing values. Given the low amount of missing data for covariates, missing values were replaced by the modal value. We used adjusted multivariable linear regression models to assess the associations between breastfeeding measures and neurodevelopmental scores. Departure from linearity for the relationship between cognitive development and continuous variables was assessed using the log‐likelihood ratio test, comparing models including the variable in tertiles as a categorical variable and as a continuous variable. When necessary, we either categorized or used restricted cubic splines. Model residuals were visually checked for normality. Variance inflation factor coefficients were estimated to detect problems of multicollinearity.

In addition, we used SEM to simultaneously analyze the relationship between the duration of breastfeeding (continuous, log‐transformed) and the cognitive tests. The WISC scores were considered as manifest variables, as were the other covariates. Due to the multidimensional structure of the NEPSY subtests, we defined one latent variable for each of the 3 domains. Regression parameters of the NEPSY and WISC variables with the duration of breastfeeding were estimated using the maximum likelihood method and adjusted by the set of potential confounders identified by the DAG.^27^ We present the relationship between variables using standardized coefficients, ranging from −1.0 for a perfect negative association to 1.0 for a perfect positive association.^28^ In certain situations (high degree of multicollinearity in the data), covariance can exceed 1.0. The model fit was assessed using the χ^2^ test (objective *P* >.05), the root mean square error of approximation (<.05) and its CI (0 to.08), the comparative fit index (>.95), the Tucker‐Lewis Index (>.9), and the goodness‐of‐fit statistic (>.9).^29^

In sensitivity analyses, we first excluded participants who had too strong an influence on the models (with Cook's distances >1). Next, we limited our analyses to participants with no imputed values for the covariates and WISC subtests. We then excluded individuals with missing breastfeeding information at 2 years old. In addition, we performed unadjusted and partially‐adjusted models that excluded the HOME score, maternal IQ, and socioeconomic status from the covariate list to fit the literature that did not adjust the analyses for maternal cognitive performance, education, and stimulation and to examine the effect of our adjustment strategy. Furthermore, we stratified the analyses of the WISC scores by deprivation index and maternal education level to observe any possible heterogeneity effects from socioeconomic background.

All analyses were performed using R software (version 3.6.1). Cubic splines were built using the splines package, and SEM analyses were performed using the Lavaan package.^27^ The results were considered statistically significant at *P* <.05.

## RESULTS

### Population Characteristics

The characteristics of the 286 woman‐child pairs studied are summarized in Table 1. The women had a median age of 30.5 years at the beginning of pregnancy, had given birth at least once prior to enrollment (57%), and had a high level of education (68% beyond a high school diploma). During pregnancy, most of them worked (91%), were nonsmokers (75%), did not drink alcohol (88%), and consumed fish less than twice a month (71%). The median birthweight was 3380 g, and 53% of the newborns were girls. The median HOME score was 46 (interquartile range [IQR], 44‐49), and 45% of the women were in the first tertile (most favorable) of the Brittany population on the Rey Socioeconomic Deprivation Index. Two‐thirds of the children were breastfed (n = 193, 67.5%), with a median duration of breastfeeding of 4 months (IQR, 2.0‐6.0). Overall, 34.9% (n = 100) were not breastfed or breastfed for less than or equal to 15 days, 34.3% (n = 98) were breastfed for 16 days to 4 months, and 30.8% (n = 88) were breastfed for at least 4 months. The results of the children's cognitive and neuropsychological test performance at 6 years of age are presented in Table 2. The median scores were 106 for the WISC‐VCI (IQR, 98‐116), 106 for the WISC‐WMI (IQR, 97‐118), and between 10 (narrative memory, 9‐11) and 14 (tower, 12‐14) for the NEPSY subtests. A comparison of our study population (N = 286) to the initially included PELAGIE cohort (N = 3421) showed the order of magnitude of the population characteristics to be similar (data not shown).

**Table 1:** Characteristics of the Study Population Stratified by Breastfeeding Status (N = 286)

Characteristics	Total Sample (N = 286)	Never Breastfed or ≤15 Days (n = 100)	Breastfed 15 Days to ≤4 Months (n = 98)	Breastfed >4 Months (n = 88)
**Characteristics of the Women**				
**Maternal age at inclusion, median (IQR), y**	30.5 (27.3‐33.2)	30.4 (27.4‐32.2)	29.1 (26.3‐33.0)	31.3 (28.6‐33.5)
**Parity at inclusion, n (%)**				
0	122 (42.7)	46 (46.0)	48 (49.0)	28 (31.8)
≥1	164 (57.3)	54 (54.0)	50 (51.0)	60 (68.2)
**Education level, n (%)** ^a^				
Less than high school diploma	42 (14.7)	25 (25.3)	12 (12.2)	5 (5.7)
High school diploma	48 (16.9)	18 (18.1)	19 (19.4)	11 (12.5)
Graduate studies	195 (68.4)	56 (56.6)	67 (68.4)	72 (81.8)
**WAIS score, median (IQR)**	93.5 (86.0‐101.0)	88.0 (82.0‐96.0)	93.0 (87.0‐101.0)	98.0 (91.8‐108.0)
**Occupational activity during pregnancy, n (%)**	259 (90.9)	91 (91.0)	92 (94.8)	76 (86.4)
**Fish consumption before pregnancy**				
Less than twice a week	204 (71.3)	74 (74.0)	72 (73.5)	58 (65.9)
Twice a week or more	82 (28.7)	26 (26.0)	26 (26.5)	30 (34.1)
**Alcohol consumption during pregnancy, n (%)** ^b^				
No	247 (87.6)	91 (92.9)	82 (84.5)	74 (85.1)
Yes	35 (12.4)	7 (7.1)	15 (15.5)	13 (14.9)
**Tobacco exposure in antenatal, n (%)** ^b^				
None	211 (74.8)	65 (66.3)	77 (79.4)	69 (79.3)
≤10 cigarettes per day	54 (19.2)	25 (25.5)	15 (15.4)	14 (16.1)
>10 cigarettes per day	17 (6.0)	8 (8.2)	5 (5.2)	4 (4.6)
**Characteristics of the Children**				
**Sex, n (%)**				
Boy	135 (47.2)	46 (46.0)	50 (51.0)	39 (44.3)
Girl	151 (52.8)	54 (54.0)	48 (49.0)	49 (55.7)
**Gestational age at birth, median (IQR), wk**	40.0 (39.0–40.0)	40.0 (39.0–41.0)	40.0 (38.2–41.0)	40.0 (39.0–40.0)
**Birth weight, median (IQR), g**	3380 (3110‐3730)	3385 (3058‐3746)	3350 (3152‐3725)	3415 (3162‐3725)
**Mode of birth, n (%)** ^c^				
Vaginal	232 (81.7)	74 (74.7)	82 (84.5)	76 (86.4)
Cesarean	52 (18.3)	25 (25.3)	15 (15.5)	12 (13.6)
**Household Characteristics**				
**HOME score, median (IQR)**	46.0 (44.0–49.0)	46.0 (43.0–49.0)	46.0 (43.2–48.0)	48.0 (45.0–50.0)
**Deprivation Rey index, n (%)** ^b^				
First tertile	126 (44.7)	36 (36.4)	42 (43.3)	48 (55.8)
Second tertile	95 (33.7)	40 (40.4)	32 (33.0)	23 (26.7)
Third tertile	61 (21.6)	23 (23.2)	23 (23.7)	15 (17.4)

Abbreviations: HOME, Home Observation for Measurement of the Environment; IQR, interquartile range; WAIS, Wechsler Adult Intelligence Scale.

a missing = 1

b missing = 4

c missing = 2

**Table 2:** Child Performances in the Study Population at 6 Years of Age for the WISC‐IV and the NEPSY Neurodevelopment Test

Outcome	n	Median (IQR)
**WISC‐IV**		
Verbal Comprehension Index^a^	286	106.0 (98.0‐116.0)
Working Memory Index^a^	282	106.0 (97.0‐118.0)
**NEPSY**		
Towers	286	14 (12‐14)
Auditory Attention and Response Set	286	11 (10‐13)
Visual Attention	284	11 (10‐13)
Design Copying	286	12 (10‐14)
Arrows	286	12 (11‐14)
Memory for Faces	286	12 (10‐13)
Memory for Names	283	10 (9‐12)
Narrative Memory	285	10 (9‐11)

WISC‐IV scores are normalized on a scale with a mean of 100 and a SD of 15 (minimum‐maximum, 40‐160). For each NEPSY subtest, the mean scaled score was 10 and the SD was 3 (minimum‐maximum, 1‐19)

Abbreviations: IQR, interquartile range; NEPSY, Developmental Neuropsychological Assessment; VCI, Verbal Comprehension Index; WISC‐IV, Wechsler Intelligence Scale for Children, fourth edition; WMI, Working Memory Index.

a Imputed data for incomplete scoring: 4 for the WISC‐VCI score and 12 for the WISC‐WMI score. Missing scores were predicted from their available subtests with linear regression models.

### Breastfeeding and Cognitive Scores

The WISC‐VCI scores were 5 points higher, on average, for children breastfed for at least 4 months than for those who were never breastfed or breastfed for less than or equal to 15 days (β_adjusted_ = 4.95; 95% CI, 0.54‐9.37), but no clear difference was observed for children breastfed for 16 days to 4 months (β_adjusted_ = −0.80; 95% CI, −4.84 to 3.24) (Table 3). Among breastfed children, the duration of breastfeeding was associated with a more statistically significant WISC‐VCI score (per log_10_ increase in months, n = 193; β_adjusted_, 8.01; 95% CI 3.31‐12.71). We did not observe a threshold effect for the relationship between the duration of breastfeeding and the WISC‐VCI score after graphical observation; nevertheless, the beneficial effect was stronger in the first 4 months of breastfeeding, with a steeper slope (Supporting Information: Appendix S3).

**Table 3:** Child Performance on NEPSY Subtests at 6 Years of Age According to Breastfeeding Status and Duration

			Domains of NEPSY							
	Cognitive Functions		Attention and Executive Functioning			Visuospatial Processing		Memory and Learning		
Characteristics	WISC‐VCI, β^A^ (95% CI)	WISC‐WMI, β^A^ (95% CI)	Towers, β^A^ (95% CI)	Auditory Attention and Response Set, β^A^ (95% CI)	Visual Attention, β^A^ (95% CI)	Design Copying, β^A^ (95% CI)	Arrows, β^A^ (95% CI)	Memory for Faces, β^A^ (95% CI)	Memory for Names, β^A^ (95% CI)	Narrative Memory, β^A^ (95% CI)
**Breastfeeding Status** ^a^										
0‐15 days (n = 100)	0 [reference]	0 [reference]	0 [reference]	0 [reference]	0 [reference]	0 [reference]	0 [reference]	0 [reference]	0 [reference]^b^	0 [reference]^c^
16 days‐4 mo (n = 98)	−0.80 (−4.84 to 3.24)	0.31 (−3.68 to 4.30)^d^	−0.20 (−0.75 to 0.35)	0.03 (−0.57 to 0.63)	−0.01 (−0.68 to 0.66)^c^	−0.05 (−0.86 to 0.75)	0.20 (−0.45 to 0.85)	−0.01 (−0.81 to 0.80)	−0.09 (−0.79 to 0.62)^c^	−0.09 (−0.73 to 0.56)
>4 mo (n = 88)	4.95 (0.54‐9.37)	0.88 (−3.43 to 5.19)^c^	0.02 (−0.58 to 0.62)	0.27 (−0.39 to 0.92)	0.30 (−0.43 to 1.03)^c^	0.55 (−0.33 to 1.43)	1.00 (0.29‐1.71)	−0.18 (−1.06 to 0.70)	0.23 (−0.54 to 0.99)	1.20 (0.50‐1.89)
**Breastfeeding Duration** ^e^										
Continuous log_10_, mo	8.01 (3.31‐12.71)	1.67 (−3.05 to 6.40)^f^	0.12 (−0.51 to 0.74)	0.43 (−0.23 to 1.08)	1.12 (0.34‐1.90)^b^	1.11 (0.15‐2.07)	0.98 (0.25‐1.72)	0.16 (−0.82 to 1.13)	0.34 (−0.51 to 1.19)^e^	*P* = 0.002^g^

β^A^ coefficient estimated by multivariable linear regression, adjusted for maternal WAIS score and education level, HOME score, birth weight, deprivation Rey index, parity, mode of birth, and occupational activity during pregnancy. For each NEPSY subtest, the mean scaled score was 10, and the SD was 3 (minimum‐maximum, 1‐19). Subtests missing data are noted.

Abbreviations: NEPSY, Developmental Neuropsychological Assessment, WAIS, Wechsler Adult Intelligence Scale; WISC‐VCI, Wechsler Intelligence Scale for Children Verbal Comprehension Index; WISC‐WMI, Wechsler Intelligence Scale for Children Working Memory Index.

a All population (N = 286).

b Missing = 2.

c Missing = 1.

d Missing = 3.

e Among children who were breastfed (n = 193; continuous analyses were restricted to the 193 children who were breastfed).

f Missing = 4.

g Positive dose‐response but nonlinear relation; model using restricted cubic splines.

There were no differences in the WISC‐WMI scores between children who were breastfed for at least 4 months and those who never breastfed or breastfed for less than or equal to 15 days (β_adjusted_, 0.88; 95% CI, −3.43 to 5.19). Similarly, there was no association using the duration of breastfeeding as a continuous variable (β_adjusted_, 1.67; 95% CI, −3.05 to 6.40) (Table 3, Supporting Information: Appendix S3).

### Breastfeeding and Neurodevelopment Scores

On average, children who breastfed for more than 4 months obtained a score that was one point higher for the arrow subtest than those who were never breastfed or were breastfed for less than or equal to 15 days (β_adjusted_, 1.00; 95% CI, 0.29‐1.71), as well as a 1.20 point higher score for the narrative memory subtest (β_adjusted_, 1.20; 95% CI, 0.50‐1.89) (Table 3). The estimated associations of neurodevelopment scores with the intermediate duration of breastfeeding (16 days to 4 months), as well as between breastfeeding status and the other NEPSY subtest, were generally close to null and not statistically significant. Among children who were breastfed, the duration of breastfeeding was associated with performance in 3 domains of the NEPSY: visuospatial attention from the attention and executive functioning domain (per log_10_ increase, in months of breastfeeding duration: β_adjusted_, 1.12; 95% CI, 0.34‐1.90), design copying and arrows from the visuospatial processing domain (β_adjusted_, 1.11; 95% CI, 0.15‐2.07; β_adjusted_, 0.98, 95% CI, 0.25‐1.72, respectively), and narrative memory from the memory and learning domain (positive but nonlinear association modeled using restricted cubic splines, overall *P* = .002) (Table 3; Supporting Information: Appendix S3). When the association was found to be statistically significant, the slopes were the steepest in the first 2 to 6 months of breastfeeding, depending on the subtest (Supporting Information: Appendix S3).

### Duration of Breastfeeding and Cognitive and Neurodevelopment Scores in SEM

**Figure 1:**
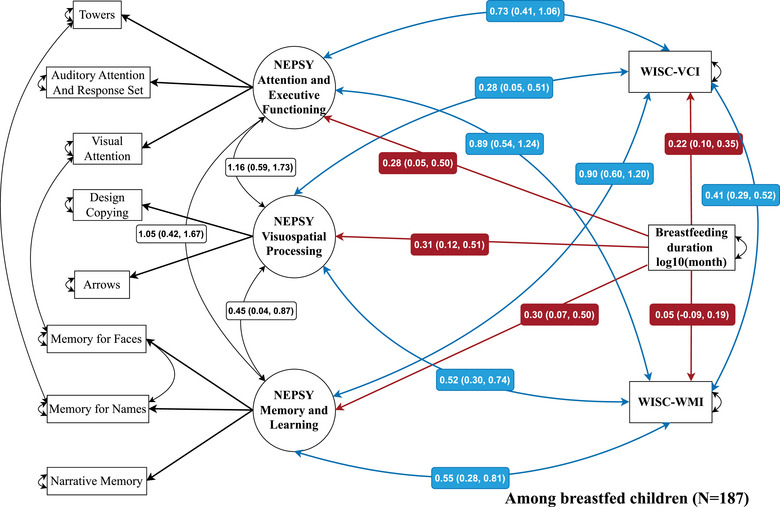
**Structural Equation Modeling of Relationships Between The Duration of Breastfeeding and Children's Neurodevelopment Test Performance at 6 Years of Age (N = 187)**. Circles denote latent variables, each representing one of the 3 domains of the NEPSY: attention and executive functioning (3 subtests: towers, auditory attention and response set, and visual attention), visuospatial processing (2 subtests: design copying and arrows), and memory and learning (3 subtests: memory for faces, memory for names, and narrative memory). Rectangles denote observed variables. Single arrows represent the direct influence of one variable upon another, and the red arrows represent relationships between latent traits of neuropsychological scores and breastfeeding. Double‐headed curvilinear arrows represent covariances between variables (blue arrows: latent traits of NEPSY and WISC score) or residual variances of observed variables. Regression coefficients indicated in the red boxes on the arrows are SEM standardized coefficients, expressed as the number of SDs of the latent trait, adjusted for maternal WAIS score and education level, HOME score, birth weight, Rey Socioeconomic Deprivation Index, parity, mode of birth, and occupational activity during pregnancy (adjustment covariates are not represented on this path diagram). WISC‐IV scores are normalized on a scale with a mean of 100 and a SD of 15 (minimum‐maximum, 40‐160). For each NEPSY subtest, the mean scaled score was 10 and the SD was 3 (minimum‐maximum, 1‐19). Fit indices: χ^2^ = 105.5, *df* = 98, *P* = 0.284; RMSEA = 0.020 (90% CI, 0.000‐0.045); CFI = 0.989; TLI = 0.981; GFI = 0.905. Abbreviations: CFI, comparative fit index; GFI, goodness‐of‐fit; HOME, Home Observation for Measurement of the Environment; NEPSY, Developmental Neuropsychological Assessment; RMSEA, root mean square error of approximation; SEM, structural equation modeling; TLI, Tucker‐Lewis Index; WAIS, Wechsler Adult Intelligence Scale; WISC‐VCI, Wechsler Intelligence Scale for Children Verbal Comprehension Index; WISC‐WMI, Wechsler Intelligence Scale for Children Working Memory Index.

In the SEM analysis, there were statistically significant positive covariances between all NEPSY latent domains and WISC scores. The duration of breastfeeding was positively and statistically significantly associated with the WISC‐VCI score and the 3 latent domains of the NEPSY after accounting for their interrelationships. Specifically, for each log_10_ increase in months of breastfeeding duration, the adjusted standardized β coefficients were as follows: WISC‐VCI score (β, 0.22; 95% CI, 0.10‐0.35), the attention and executive functioning domain (β, 0.28; 95% CI, 0.05‐0.50), the visuospatial processing domain (β, 0.31; 95% CI, 0.12‐0.51), and the memory and learning domain (β, 0.30; 95% CI, 0.07‐0.50) (Figure 1). We did not find any statistically significant association between the duration of breastfeeding and the WISC‐WMI scores (β_adjusted_, 0.05; 95% CI, −0.09 to 0.19). The fit statistics were satisfactory for all models (see legend of Figure 1).

### Sensitivity Analysis

Associations between the children's cognitive performance and breastfeeding did not change in the various sensitivity analyses, except when restricting our analyses to the population with breastfeeding information at age 2 years (n = 198); the arrow subtest became nonstatistically significant, but the adjusted coefficients remained similar in magnitude and direction (data not shown). No influential individual was detected by a Cook's distance greater than 1. In crude and partly adjusted models, we observed a stronger association between WISC‐VCI and breastfeeding status (up to 2‐fold) and duration than in the fully‐adjusted model (Supporting Information: Appendix S4).

Stratifying results based on the Rey Socioeconomic Deprivation Index showed a threefold higher effect of the duration of breastfeeding on the WISC‐VCI scores among children living in the most deprived area than those living in the least deprived one (Supporting Information: Appendix S5). Only analyses studying the association between the duration of breastfeeding and WISC‐VCI scores showed statistically significant associations for the 2 lowest (most deprived) tertiles. We observed similar trends for lower maternal education levels (data not shown).

## DISCUSSION

In our study, a longer duration of breastfeeding was associated with higher performance in one neurocognitive domain (WISC‐VCI) and several subtests of 3 NEPSY neuropsychological domains (attention and executive functioning, visuospatial processing, and memory and learning) among 6‐year‐old children. We did not observe a threshold effect but noted that the slope in terms of cognitive gain was the steepest for the first 2 to 6 months of breastfeeding. SEM analysis confirmed the associations between the various cognitive scores and breastfeeding after considering the interrelation between them.

Previous studies conducted in high‐income countries have shown similar positive associations of verbal ability (WISC‐VCI) with the duration of breastfeeding, but the literature is somewhat dated, with subsequent changes in clinical practices and breastfeeding management.^11^, ^30^ However, these previous findings were not adjusted for maternal IQ. A meta‐analysis by Horta et al^3^ suggested that this was an important confounder because breastfeeding behavior and child development are influenced by maternal intelligence. Indeed, adjustment for this parameter tended to reduce the benefit of breastfeeding on cognitive performance.^3^ The role of socioeconomic status also appears to be predominant according to studies that focused on the causal relationship between breastfeeding and cognitive development.^31^, ^32^, ^33^ The results have been more heterogeneous for studies that adjusted for these parameters. Belfort et al^34^ and Horta et al^2^ observed positive associations, whereas 3 other studies also conducted in high‐income countries did not.^16^, ^17^, ^35^ The coefficient parameter was indeed reduced (divided by 2) between our partially‐adjusted and fully‐adjusted models (additionally adjusted for woman's IQ, woman's education level, and HOME score), but the results remained statistically significant.

The heterogeneity of the results in the literature can be explained by differences in the definition of breastfeeding between studies (exclusive or not, duration, and status [yes or no]), as well as in the study populations and tests used to assess the children's cognitive development (differences in sensitivity).^1^, ^32^ Nevertheless, Horta et al considered that such differences between studies could not themselves explain such heterogeneity.^2^ Indeed, several studies were conducted in low‐income or middle‐income countries, where breastfeeding is associated with a less favorable socioeconomic context. These studies also tended to report a positive association between breastfeeding and neurodevelopment, suggesting an effect of breastfeeding independent of the maternal socioeconomic status.^7^, ^31^

Focusing on nonverbal IQ, we found nonsignificant associations between breastfeeding and the WISC‐WMI scores, which tended to disappear with adjustment. Sajjad et al obtained the same results;^17^ the benefit of breastfeeding on nonverbal IQ was mainly explained by the woman's IQ and education level, as well as the home family stimulation. Only 2 studies conducted by Belfort et al and Pérez Ruiz et al that accounted for the woman's IQ reported significantly higher scores for nonverbal IQ.^34^, ^36^ However, we observed a significant positive association between breastfeeding and a subtest involving memory in the memory and learning domain of the NEPSY. Compared with the cognitive functions related to IQ, the relationship between breastfeeding and child neuropsychological development has been poorly studied. A previous study suggested an increased risk of neuropsychological deficits during adolescence and early adulthood when breastfeeding was of shorter duration.^37^ Others have suggested a statistically significant association between the duration of breastfeeding for all types of breastfeeding^38^ or exclusive breastfeeding^39^ and the neuropsychological performance of the child, whereas another did not observe a statistically significant association with breastfeeding^40^ or exclusive breastfeeding.^18^, ^41^ These conflicting results can be explained by differences in study design, sample size, the neurodevelopmental assessments used, and adjustment strategy.

We used SEM analysis to integrate the complex relationship between all cognitive scores in analyzing their association with breastfeeding. SEM allows the simultaneous study of several outcomes, taking into account multicollinearity and the interrelation between variables. The associations between the various neurodevelopment scores and breastfeeding found in the main analysis remained statistically significant after considering the relationship between them in the SEM approach, reinforcing our confidence in the results.

Our results report a stronger association between the duration of breastfeeding and the WISC scores among children living in areas with a higher Rey Socioeconomic Deprivation Index than those living in areas with a lower Rey Socioeconomic Deprivation Index. This result is in accordance with that of the study of McCrory et al, which found that breastfeeding had greater effects on the reading and mathematical test scores of socially disadvantaged nine‐year‐olds than children from more advantaged social groups in an Irish cohort study.^42^ In other situations, such as those of premature infants, who may be considered to be at higher risk of lower cognitive test results, a more striking effect of breastfeeding on cognitive development was observed than for term infants, even when the duration of breastfeeding was short (<2 months).^13^ These results suggest that breastfeeding could be part of a strategy to compensate for adverse environmental stressors for the most vulnerable newborns. Implementing breastfeeding support programs for families in precarious situations is crucial, especially as breastfeeding rates are lower in such families than those with higher education levels. Such programs would allow compensation, at least in part, for the health impairment of children associated with social disadvantage. The benefits of breastfeeding, including enhanced neurodevelopment, could possibly contribute to helping break the cycle of poverty, given its proven effectiveness.^7^

Several mechanisms have been suggested to explain the influence of breastfeeding on child neurodevelopment, but there is currently no clear consensus and no identified specificity concerning the cognitive domain. However, a recent study has provided evidence of the mechanism by which breastfeeding may have a positive effect on brain development and, consequently, on cognitive development and mental health in children at 8 years of age.^43^ Human milk contains higher concentrations of essential long‐chain polyunsaturated fatty acids (PUFAs), such as arachidonic acid, docosahexaenoic acid, and oligosaccharides, than human milk substitutes. These PUFAs influence the thickness of the parietal cortex and may play a role in neonatal brain development.^44^, ^45^ Gene‐environment interactions have been suggested, with *FADS* genes encoding fatty acid desaturase, suspected of modulating the relationship between breastfeeding, exposure to PUFAs, and children's cognition.^46^ Other mechanisms have been hypothesized, such as a synergy between bioactive substances in human milk that regulate the maturation of the intestinal mucosal barrier, which could influence neurobiological development through the gut‐brain axis from early childhood.^47^ Nevertheless, the few studies that have focused on PUFA supplementation did not demonstrate any statistically significant impact of such supplementation on cognitive development.^35^ Another hypothesis focuses on the woman's interaction with her child, with mothering and woman‐child bonding enhanced by the simple practice of breastfeeding, which may favor the future sociocultural level of the child,^16^, ^33^, ^48^ or even through all these components at once. A woman's decision to breastfeed, particularly for an extended period, reflects a safe and sound maternal attachment status, which has been shown to have a positive influence on the child's psychological development into later age.^49^ Nonetheless, after controlling for such direct measures of parental input in our study, breastfeeding itself and its duration remained statistically beneficial for the child's cognitive abilities.

Four prospective birth cohort studies conducted in Brazil, the United Kingdom, and Denmark found a positive effect of breastfeeding on school achievement and an association between breastfeeding and intellectual performance a few years later in adulthood, showing a positive impact on education attainment and adult income.^8^, ^9^, ^15^, ^50^ According to these results, the effect of breastfeeding on IQ at 6 years of age in our study could persist over the long term, emphasizing its promotion to guide national public health policies. A woman's knowledge can influence her intention to breastfeed; providing information to pregnant women on the benefits of breastfeeding can affect the decision for those who have not already made a choice.^49^ Our results support the WHO's global targets of 2025,^4^ which aim to increase the initiation rate of breastfeeding and its duration through various actions.

Our study had several limitations. Breastfeeding data were collected retrospectively at the ages of 2 (69%) and 6 years (31%). A previous study suggested that women who breastfed for short periods tended to overreport the duration, whereas those who breastfed longer tended to underreport.^51^ Such misclassification, which is likely nondifferential between groups, may have led to an attenuation of the associations, bringing them closer to the null.^51^ In addition, when we restricted our study population to children with available information at age 2, we noted that the coefficients remained similar in magnitude and direction. Exclusive breastfeeding was not assessed in the PELAGIE cohort. Several studies have reported a stronger association between the duration of exclusive breastfeeding and cognitive development assessments than for the duration of any type of breastfeeding (mixed or not), which tended to exclude false positives due to such misclassification.^3^, ^11^, ^13^, ^31^, ^52^

Although the PELAGIE cohort is not representative of the French population, we observed a similar rate and duration of breastfeeding in the French representative nationwide cohort (70% breastfeeding vs 67% in PELAGIE; median duration, 3.9 months vs 4.0 months, respectively).^49^ Moreover, the 286 woman‐child pairs who agreed to participate were similar to the population at inclusion, which indicates a limited influence of attrition bias on our results.

This is the first study conducted in France showing the relationship between breastfeeding and cognitive performance controlling for maternal IQ. In addition, our stratified analysis of the deprivation index highlights the benefits of encouraging breastfeeding to help reduce social inequalities among children from less‐privileged backgrounds. In light of our results, it would be informative to explore the effect of exclusive breastfeeding on child neurodevelopment while also considering the father's education level.^35^

Our study had other strengths, including the prospective design with numerous potential confounders collected during the study, including several markers of individual and/or contextual socioeconomic level (woman's verbal IQ and education level, HOME score, Rey Socioeconomic Deprivation Index), using several validated neurocognitive and neurobehavioral assessments. Furthermore, the associations found were consistent using 2 statistical approaches (multivariable linear regression and SEM) and in multiple sensitivity analyses. Another strength of this study was the use of SEM analyses, which allowed the study of complex interrelations between scores that have rarely been studied together.

## CONCLUSION

In conclusion, our study conducted in a French population‐based cohort supports an association between breastfeeding and verbal comprehension WISC scores at age 6, as well as certain subtests of the NEPSY scale. Further research should be considered to further investigate the mechanisms through which human milk affects child neurodevelopment. Improved health policy and legislation on maternity and paternity leave, as well as adapted workplace policies, could help increase the duration of breastfeeding.

## CONFLICT OF INTEREST

The authors have no conflicts of interest to disclose

## Supporting information




**Appendix S1**. Strengthening the Reporting of Observational Studies in Epidemiology (STROBE) checklist
**Appendix S2**. Directed Acyclic Graph
**Appendix S3**. Relationship Between the Duration of Breastfeeding and Performance on Neurodevelopmental Tests of Children at 6 Years of Age Among Children Who Were Breastfed
**Appendix S4**. Child Cognitive Function at 6 Years of Age According to Breastfeeding Status (All Children) and Duration (Among Children Who Were Breastfed)
**Appendix S5**. Relationship Between Breastfeeding (Status and Duration) and the WISC Verbal Comprehension and Working Memory Indexes at Six Years of Age, Stratified by the Rey Deprivation Index and Education Level Categories (Adjusted Coefficients (95% CI)

Supporting Information
