# Supplementary material for: Association Between Breastfeeding and Neurodevelopment at 6 Years of Age in the French PELAGIE Birth Cohort
Source: J Midwifery Womens Health. 2025 Jun 3;70(4):640–50. doi: 10.1111/jmwh.13766 (PMC12365730; doi:10.1111/jmwh.13766)
Supplement: Supplementary file 2 — Supporting Information [file JMWH-70-640-s002.docx]

# **Supporting Information: Appendices**

# Appendix S2. DAGitty output (<http://dagitty.net>): directed acyclic graph representing the relationships between exposure (breastfeeding), outcome (neurodevelopment), and related factors.


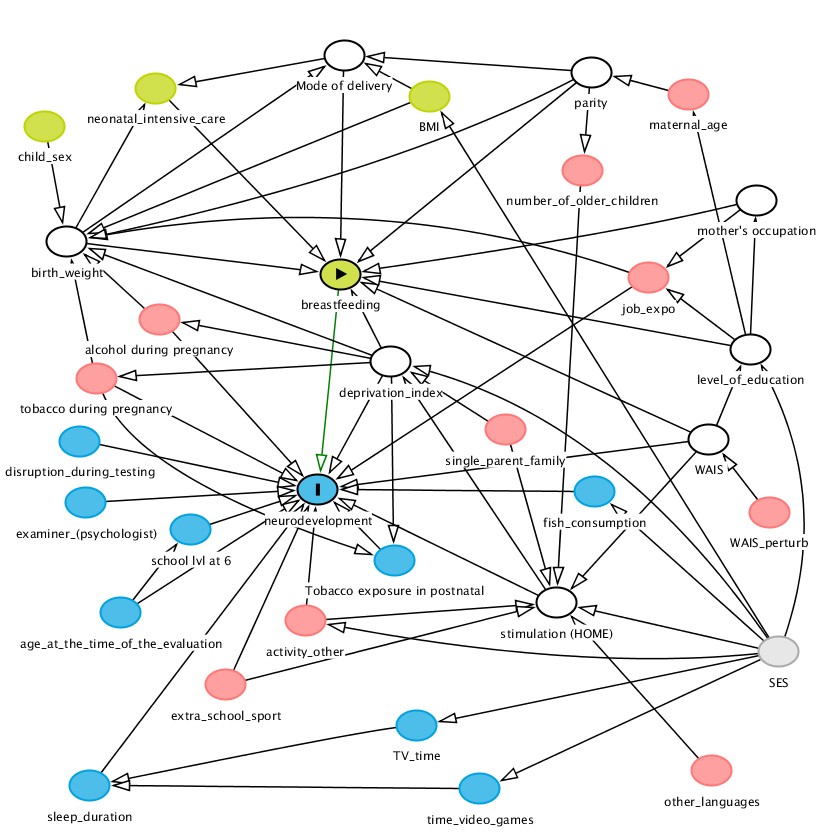


We used the same set of covariates in all statistical models: mother’s verbal IQ (WAIS), education level, HOME score, infant birth weight, Rey Socioeconomic Deprivation Index, parity, mode of delivery, and occupational activity during pregnancy.

# Appendix S3. Relationship between the duration of breastfeeding and performance on neurodevelopmental tests of children at six years of age among breastfed children

#
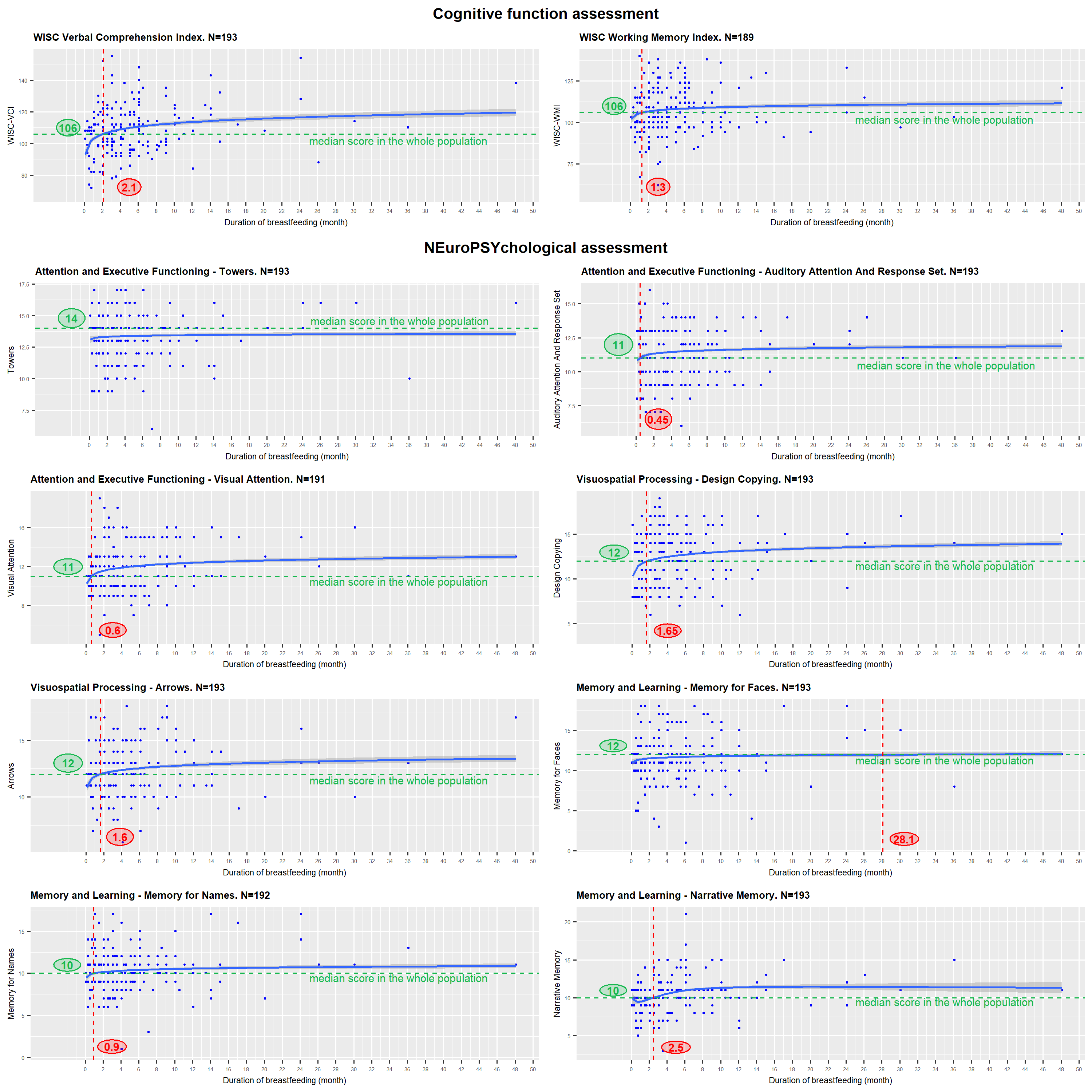


Graph of the predicted WISC and NEPSY values based on a multivariable linear regression adjusted model fitted by log transformation of the duration of breastfeeding (blue, continuous line) and the 95% confidence interval (dark grey, shaded area) and scatter plot of the actual values of the WISC and NEPSY scores according to the duration of breastfeeding (blue, points). The median WISC and NEPSY score for the whole population (N=286 for WISC-VCI; N=282 for WISC-WMI; 283, 284 or 286 for NESPSY) is represented by the green dotted line. The red dotted line corresponds to the intersection between the curve of the predicted WISC or NEPSY values and the line representing the median WISC or NEPSY score for the whole population. The Y-axis represents the change in the WISC or NEPSY score. The X-axis represents the duration of breastfeeding in months. Each graph corresponds to one cognitive function (WISC-VCI or WISC-WMI) or one subset of a NEPSY domain. For the Narrative Memory subset, we used restricted cubic splines (natural splines as knots) because of non-compliance with the linearity assumption.

Adjusted for maternal WAIS score and education level, HOME score, birth weight, Rey Socioeconomic Deprivation Index, parity, mode of delivery, and occupational activity during pregnancy.

Abbreviations: NEPSY, Developmental Neuropsychological Assessment; WISC-VCI, Wechsler Intelligence Scale for Children Verbal Comprehension Index; WISC-WMI, Wechsler Intelligence Scale for Children Working Memory Index.

WISC-IV scores were normalized on a scale with a mean of 100 and a standard deviation of 15 (minimum–maximum, 40–160)

For each NEPSY subtest, the mean scaled score was 10 and the standard deviation was 3 (minimum–maximum, 1–19

#

# Appendix S4. Child cognitive function at six years of age according to breastfeeding status (all children) and duration (among breastfed children)

|  | **WISC-Verbal Comprehension Index** | | | | **WISC-Working Memory Index** | | | |
| --- | --- | --- | --- | --- | --- | --- | --- | --- |
|  | **N** | **β_crude_ (95% CI)** | **β_partly adjusted_^c^ (95% CI)** | **β_adjusted_^d^ (95% CI)** | **N** | **β_crude_ (95% CI)** | **β_partly adjusted_^c^ (95% CI)** | **β_adjusted_^d^ (95% CI)** |
| **Breastfeeding status^a^** | **286** |  |  |  | **282** |  |  |  |
| 0 - 15 days | 100 | 0 [reference] | 0 [reference] | 0 [reference] | 100 | 0 [reference] | 0 [reference] | 0 [reference] |
| 16 days - 4 months | 98 | 1.27 (-2.91, 5,44) | 1.09 (-3.08, 5.27) | -0.80 (-4.84, 3.24) | 95 | 1.39 (-2.62, 5.41) | 1.30 (-2.74, 5.34) | 0.31 (-3.68, 4.30) |
| > 4 months | 88 | 9.10 (4.81, 13.39) | 9.11 (4.73, 13.49) | 4.95 (0.54, 9.37) | 87 | 4.62 (-0.51, 8.72) | 3.57 (-0.64, 7.79) | 0.88 (-3.43, 5.19) |
| **Breastfeeding duration^b^** | **193** |  |  |  | **189** |  |  |  |
| Continuous, log10(month) |  | 9.82 (5.09, 14,55) | 10.07 (5.33, 14.81) | 8.01 (3.31, 12.71) |  | 3.52 (-1.09, 8.14) | 2.66 (-2.09, 7.41) | 1.67 (-3.05, 6.40) |

^a^ Entire population

^b^ Among breastfed children (continuous analyses were restricted to the 193 breastfed children)

^c^ Adjusted for birth weight, deprivation Rey index, parity, mode of delivery, and occupational activity during pregnancy

^d^ Adjusted for maternal WAIS score and education level, HOME score, birth weight, Rey Socioeconomic Deprivation Index, parity, mode of delivery, and occupational activity during pregnancy

^e^ missing = 0; ^f^ missing = 1; ^g^ missing = 3; ^h^ missing = 4;

β Coefficient estimated by multivariable linear regression.

Abbreviations: WISC, Wechsler Intelligence Scale for Children; CI, confidence interval.

WISC-IV scores were normalized on a scale with a mean of 100 and a standard deviation of 15 (minimum–maximum, 40–160)

Appendix S5. Relationship between breastfeeding (status and duration) and the WISC Verbal Comprehension and Working Memory Indexes at six years of age stratified by the Rey deprivation index and educational level categories (adjusted coefficients (95% CI))


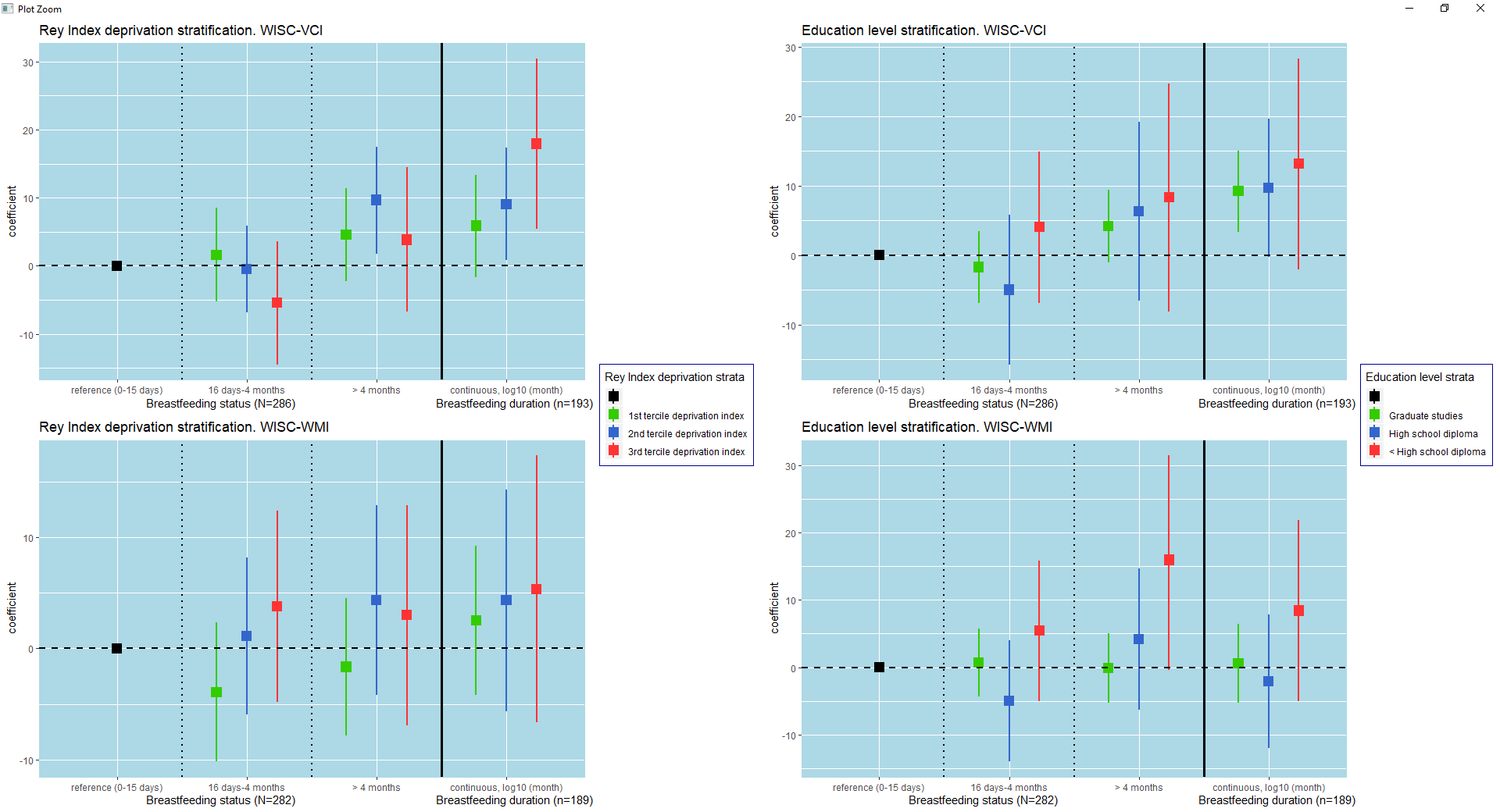


Abbreviations: WISC-VCI, Wechsler Intelligence Scale for Children Verbal Comprehension Index; WISC-WMI, Wechsler Intelligence Scale for Children Working Memory Index.

Adjusted for maternal WAIS score and education level (except if stratified by education level), HOME score, birth weight, Rey Socioeconomic Deprivation Index (except if stratified by Rey index), parity, mode of delivery, and occupational activity during pregnancy.

WISC-IV scores were normalized on a scale with a mean of 100 and a standard deviation of 15 (minimum–maximum, 40–160)
